# Turbot (*Scophthalmus maximus*) vs. VHSV (Viral Hemorrhagic Septicemia Virus): A Review

**DOI:** 10.3389/fphys.2016.00192

**Published:** 2016-05-26

**Authors:** Patricia Pereiro, Antonio Figueras, Beatriz Novoa

**Affiliations:** Instituto de Investigaciones Marinas, Consejo Superior de Investigaciones CientíficasVigo, Spain

**Keywords:** turbot, VHSV, disease, immunity, antiviral, transcriptome, resistance, QTLs

## Abstract

Turbot (*Scophthalmus maximus*) is a very valuable fish species both in Europe and China. The culture of this flatfish is well-established but several bacteria, viruses, and parasites can produce mortality or morbidity episodes in turbot farms. Viral Hemorrhagic Septicemia Virus (VHSV) is one of the most threatening pathogens affecting turbot, because neither vaccines nor treatments are commercially available. Although the mortality in the turbot farms is relatively low, when this virus is detected all the stock have to be destroyed. The main goals that need to be improved in order to reduce the incidence of this disease is to know what are the strategies or molecules the host use to fight the virus and, in consequence, try to potentiate this response using different ways. Certain molecules can be selected as potential antiviral treatments because of their high protective effect against VHSV. On the other hand, the use of resistance markers for selective breeding is one of the most attractive approaches. This review englobes all the investigation concerning the immune interaction between turbot and VHSV, which until the last years was very scarce, and the knowledge about VHSV-resistance markers in turbot. Nowadays, the availability of abundant transcriptomic information and the recent sequencing of the turbot genome open the door to a more exhaustive and profuse investigation in these areas.

## Turbot aquaculture

Turbot (*Scophthalmus maximus*) is an economically relevant flatfish species belonging to the family Scophthalmidae (order Pleuronectiformes) widely distributed from Norway to the Mediterranean and the Black Sea (Nielsen, 1986). The first steps in the production of this fish were conducted in Scotland (UK) during the 1970's, but then the turbot aquaculture was quickly expanded to Spain and France (FAO). After numerous technical and biological improvements, their production was also initiated in other European countries (Portugal, Denmark, Germany, Iceland, Ireland, Italy, Norway, and Wales). Nowadays, the culture of this fish is well-established and the complete farm-raising cycle is conducted in land-based aquaculture facilities.

In Europe, turbot aquaculture production was about 11,000 tonnes in 2014, a 38.3% higher with regard to 2013, being Spain the main European producer [Asociaci3n Empresarial de Productores de Cultivos Marinos (APROMAR), 2015]. Indeed, 7808 tonnes were produced in Spain in 2014. This species, native to Europe, is also cultured in Chile (about 107 tonnes per year) but especially in China, reaching an annual level of 50,000–60,000 tonnes in recent years and being the largest producer of turbot in the world [Food and Agriculture Organization of the United Nations (FAO), 2010]. Currently in Spain only a third of the turbot in the markets comes from fisheries [Asociaci3n Empresarial de Productores de Cultivos Marinos (APROMAR), 2015].

Nevertheless, there are still some limitations affecting the culture of this flatfish, such as the low genetic renewal of the breeders and specific diseases which causes mortality and morbidity episodes, with the subsequent economic losses. The development of the turbot aquaculture caused a concomitant increase in pathological conditions affecting the culture of this flatfish. Several pathogens, including bacteria, viruses, and parasites affect the health status of the farmed turbot. Although, today there are effective treatments or vaccines available against a variety of pathogens affecting turbot culture, other diseases, especially those induced by viral agents, have not an easy solution. Neither vaccines nor therapeutic treatments are commercially available for the most of the viral diseases affecting fish.

Viruses are probably the most destructive pathogens encountered in aquaculture and they are a serious concern since no specific chemotherapies are available. To illustrate the impact of fish viruses it can be pointed out that among the 10 notifiable fish diseases (diseases with great social and economic and/or public health repercussion or with present or potential risk for the aquaculture industry) appearing at the 2014 Aquatic Animal Health Code of the OIE (Office International des Epizooties, now the World Organization for Animal Health) (http://www.oie.int), eight are caused by viruses. Viral Hemorrhagic Septicemia Virus (VHSV), a virus affecting *S. maximus* production, is included in this OIE list.

## Viral hemorrhagic septicemia virus (VHSV)

### Taxonomy, structure, genotypes, and geographical distribution

**Figure 1:**
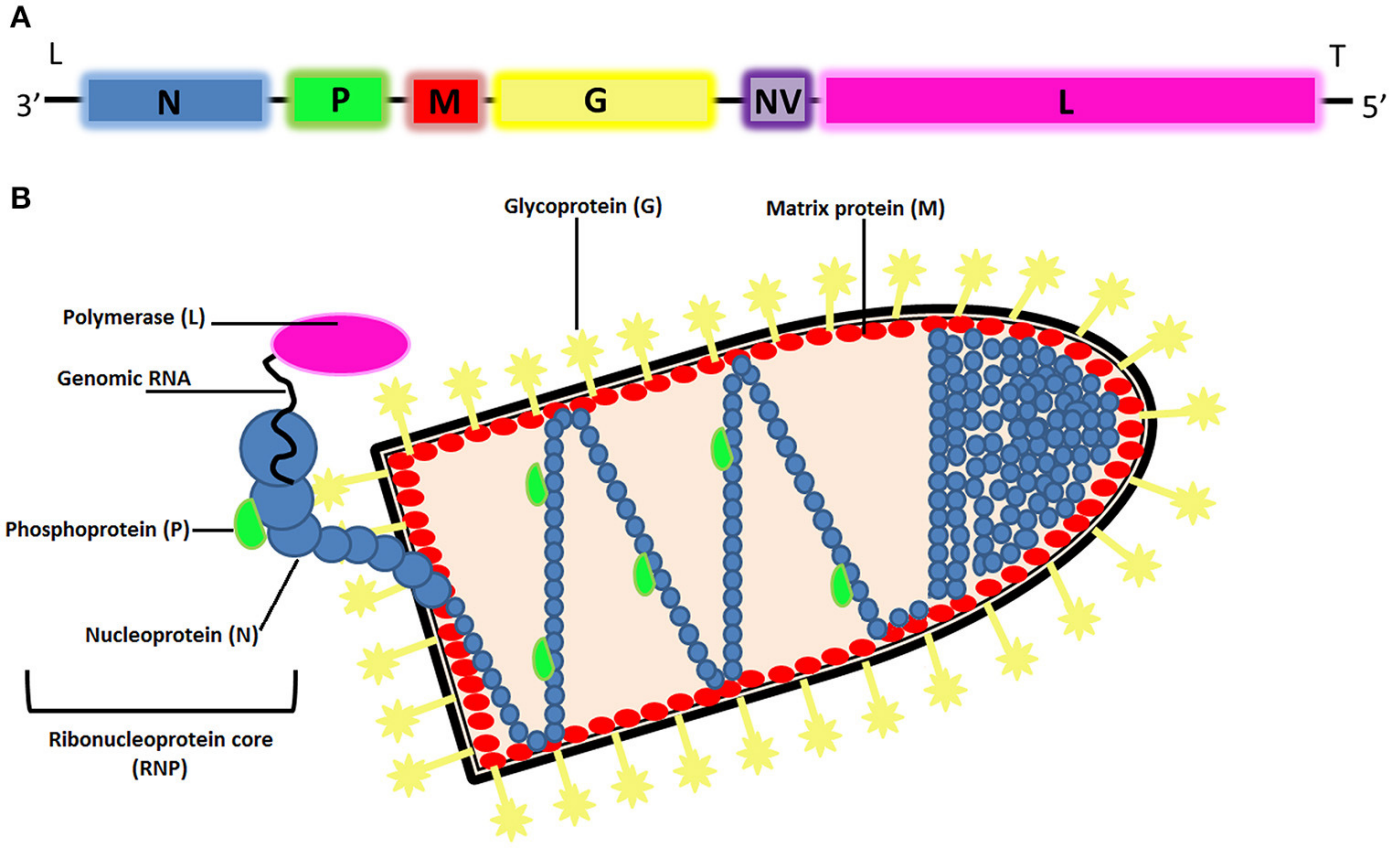
**(A)** Organization of the VHSV genome. The gene order of VHSV is 3′-leader-N-P-M-G-NV-L-trailer-5′. **(B)** Schematic representation of the morphology and structural components of rhabdoviruses.

VHSV is a fish pathogen belonging to the genus Novirhabdovirus, within the family Rhabdoviridae (Walker et al., 2000; Tordo et al., 2005). Rhabdoviruses are bullet-shaped, 170–180 nm in length, and 60–70 nm in width (Elsayed et al., 2006), enveloped viruses with a simple negative-sense, single-stranded RNA (ssRNA) genome of ~11 kb (Schutze et al., 1999). The typical rhabdoviral genome encodes five basic structural proteins: nucleoprotein (N), polymerase-associated phosphoprotein (P), matrix protein (M), glycoprotein (G), and large RNA-dependent RNA polymerase (L). Members of the genus Novirhabdovirus are distinguished by the presence a sixth gene located in the genome between the G and the L genes, encoding a non-structural or non-virion (NV) protein (Kurath and Leong, 1985; Schutze et al., 1999), which is implicated in the pathogenesis (Ammayappan and Vakharia, 2011; Choi et al., 2011; Figure 1A). All rhabdoviruses possess non-coding 3′ leader and 5′ trailer sequences.

Structurally, all rhabdoviruses have two major structural components: a helical ribonucleoprotein core (RNP) and a surrounding envelope (Figure 1B). In the RNP, genomic RNA is tightly encased by the nucleoprotein. The phosphoprotein and the large protein (L-protein or polymerase) are also associated with the RNP. The glycoprotein (G) forms trimeric spikes which are tightly inserted into the lipid bilayer (typical of enveloped viruses and derived from portions of the host cell membranes). Under and associated to the membrane by hydrophobic and electrostatic interactions is a layer formed by the matrix protein (M), which condenses the RNP. Moreover, M protein is also associated with the lipid bilayer and the glycoprotein, forming a link between the ribonucleocapsid and glycoproteins in the viral envelope (Assenberg et al., 2010).

Phylogenetic analysis allowed the identification of four geographically distinct major VHSV-genogroups based on N- and G-gene nucleotide variation (Snow et al., 1999, 2004; Einer-Jensen et al., 2004). Genotype I is composed of rainbow trout freshwater isolates (Genotype Ia) and marine isolates from the Baltic Sea (Ib) closely related to those belonging to Ia (Snow et al., 1999). European marine strains are divided into two groups: Baltic Sea isolates (Genotype II), and isolates from the North Sea and European Atlantic (Genotype III). Finally, Genotype IV is composed by North America strains. Additionally, Genotype I and IV can also be divided into five (Ia-Ie) and three (IVa-IVc) subtypes based on their reactivity to different monoclonal antibodies (Ito et al., 2012). At this regard, genotypes Ia and II revealed low mortality in experimentally infected turbot, while Ib showed an intermediate effect and the highest mortality levels were obtained in turbot infected with isolates from the Genotype III (Snow et al., 2005). Interestingly, it was observed that the differences in virulence among phylogenetically distinct VHSV isolates are not explained by the variability of the surface glycoprotein G or the non-virion (NV) protein (Einer-Jensen et al., 2014). The outbreaks detected in turbot farms were mainly caused by the UK-860/94 strain (Genotype III). Indeed, this strain was isolated from the outbreak at the Gigha turbot farm (Scotland; Ross et al., 1994).

### Fish species affected by VHSV, clinical signs, and problems in turbot farms

This etiological agent causes an important viral disease affecting rainbow trout *Oncorhyncus mykiss* and other salmonids (Castric and de Kinkelin, 1980; Hørlyck et al., 1984; Wolf, 1988) but VHSV outbreaks have been detected in other farmed fish species such as turbot (Schlotfeldt et al., 1991; Ross et al., 1994). Indeed, since the last 1970's, VHSV has been isolated from at least 50 species of marine and freshwater fish (Skall et al., 2005). It was also observed that some VHSV strains are able to infect several host species and sporadically cross species barriers (Schönherz et al., 2013).

**Figure 2:**
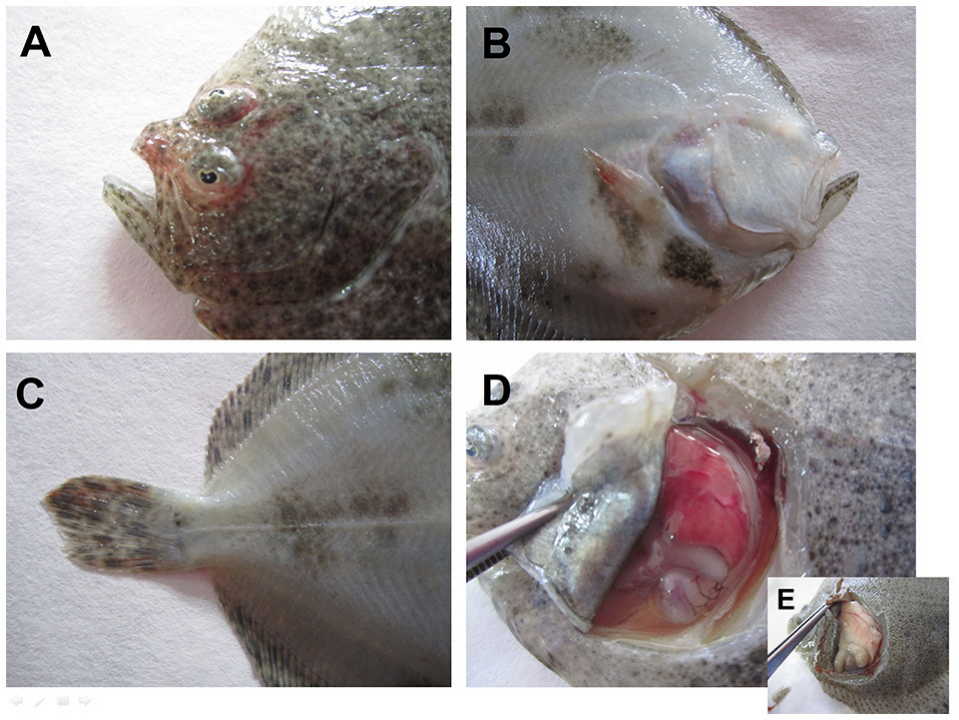
**Clinical signs in juvenile turbot intraperitoneally infected with VHSV strain UK-860/94**. External hemorrhages are observed around the eyes **(A)**, mouth **(A)**, and fins **(B,C)**. Internal organs also show severe hemorrhages, especially noticeable in the liver **(D)** when is compared with a healthy one **(E)**.

Diseased fish may display non-specific clinical signs in the early stages of infection, including rapid onset of mortality (which can reach up to 100% in fry), lethargy, darkening of the skin, exophthalmia, anemia (pale gills), hemorrhages at the base of the fins, gills, mouth, eyes, and skin, a distended abdomen due to edema in the peritoneal cavity, and severe abnormal swimming behavior. Some of the clinical signs observed after the intraperitoneal injection of the VHSV strain UK-860/94 in juvenile turbot are shown in Figure 2. Regarding to the latency of the virus, there are contradictory studies, some of them suggesting that VHSV is not able to remain in surviving fish (Snow and Smail, 1999; Chico et al., 2006; Duesund et al., 2010), whereas other investigations indicate the contrary (Schönherz et al., 2013). Nevertheless, the persistence of VHSV in the host is probably conditioned by the specificity/virulence of the VHSV genotype to the fish species and also by the lapse of time after the exposition to the virus, among other conditions. On the other hand, the different sensibility of the detection methods could provide opposite results.

In recent years, due to the intensive farming conditions, disease outbreaks caused by sea water viral pathogens have frequently become severe problems faced by turbot industries worldwide. VHS outbreaks in turbot facilities were reported from a research institution in Germany in 1991 (Schlotfeldt et al., 1991), and turbot farms in Scotland in 1994 (Ross et al., 1994), and Ireland in 1997 (Skall et al., 2005). Although no official data reporting the incidence of VHSV in European turbot farms during the last two decades are available, we cannot discard the presence of this virus in the facilities and even the possibility that VHSV could be the causative agent of punctual mortality episodes. Unfortunately, when private economic interests are implicated, the transparency is not as good as could be expected.

In the case of the first declared VHSV outbreak in a turbot farm (Ross et al., 1994), although the mortality rate was relatively low (6%), all the stock (about 14 tonnes) was collected and sacrificed as a part of a contingency plan (Hastein et al., 1999). Nowadays the European council directive 2006/88/EC lists the VHS as a non-exotic disease and, as consequence, when VHSV is detected in a fish farm the clinically healthy animals are allowed to continue growing until reaching the commercial size if no disease outbreak appears. Nevertheless, severe control measures have to be taken when a VHSV outbreak occurs in order to avoid the spread of the disease and to eradicate the virus from the facilities.

### Control and prevention

Due to the absence of effective antiviral treatments, prevention is the critical point in the eradication of this disease. In addition to the biosecurity measures that should be taken in order to prevent the entry/spread of the virus, some immune-related aspects are the key points in order to reduce the impact of viral diseases. The main prophylactic measures include probiotics, immunostimulation, and vaccination. The use of probiotics and immunostimulants in aquaculture, mainly provided through specific dietary manipulation, could help to enhance the innate immune system of fish (Olafsen, 2001; Magnadottir, 2010). Although, these substances were mainly tested against pathogen bacteria, some studies revealed their positive effect against viral diseases in teleost fish (Jensen et al., 2002; Jorgensen et al., 2003; Lockhart et al., 2004; Fernandez-Trujillo et al., 2008; Das et al., 2009; Kim et al., 2009; Son et al., 2009; Chiu et al., 2010; Harikrishnan et al., 2010; Liu et al., 2012). It was observed that the administration of DNA CpG motifs (plasmid pMCV1.4) in turbot was able to reduce the mortality caused by a VHSV challenge even 1 month after the immunostimulation (Pereiro et al., 2012c), and microarray analysis showed that some gene modulation remained active in these fish (Pereiro et al., 2014a).

No vaccines are commercially available for VHSV. For more than 30 years, an increased effort was performed in order to produce an efficient, safe, and cost-effective vaccine against VHSV using subunits or single viral proteins as well as killed or attenuated viruses (de Kinkelin et al., 1980, 1995; Bernard et al., 1983; Leong and Fryer, 1993; Lecocq-Xhonneux et al., 1994; Adelmann et al., 2008). Although, some of these vaccines had induced good protection levels in laboratory conditions, sometimes they can be unsafe for field use, its production might be very expensive or high doses may be required. Deoxyribonucleic acid (DNA) vaccination is based on the administration of a plasmid DNA vector containing the gene encoding a specific antigen. This technology is a powerful tool for the design of effective vaccines against fish pathogens. It has become clear that one of the most efficient methods for induction of a protective immune response under experimental conditions in rainbow trout against VHS and other Rhabdoviruses is DNA vaccination, being vaccines encoding viral membrane glycoproteins remarkably efficacious (Anderson et al., 1996; Winton, 1997; Lorenzen et al., 1998, 2000; LaPatra et al., 2001). Rhabdoviruses possess a surface glycoprotein (G protein) that acts as the target of virus neutralizing antibodies (Lorenzen et al., 1990) and the more successful DNA vaccines against these viruses are based on the G glycoprotein gene under the control of the cytomegalovirus promoter (CMV). Intramuscular administration of micrograms amounts of plasmid is enough for the expression of the viral G glycoprotein on the surface of muscular cells and this is the way to trigger the immune response (Lorenzen et al., 2005; Lorenzen and LaPatra, 2005). In turbot, a DNA vaccine encoding the G glycoprotein from VHSV UK-860/94 strain was tested in juvenile turbot, and highly promising results were obtained, with a relative percentage of survival (RPS) over 80% (Pereiro et al., 2012c). Moreover, the transcriptome analysis after the administration of this vaccine showed an activation of the main immune-pathway in a similar way to that induced by VHSV (Pereiro et al., 2014a). One month after vaccination, the transcriptome profile was totally different among vaccinated and non-vaccinated individuals, showing this last group an uncontrolled and intense innate immune response (Pereiro et al., 2014a). These results reflect the potential of the DNA vaccination strategies in fish aquaculture. Nevertheless, fish immunization by an antigen-encoding DNA vaccine was only approved for commercial sale in Canada against IHNV (also a Novirhabdovirus) in farmed salmon (Evensen and Leong, 2013). At present there has not been approved any DNA vaccine to be used in aquaculture in Europe.

Another way to prevent, or al lest reduce, the prevalence of one disease is the genetic improvement. Marker-assisted selection (MAS) in fish breeding schemes has become a very promising strategy for obtaining individuals with a certain traits of interest. In fish aquaculture these traits are specially focused on growth, sex determination, and resistance to diseases. Nowadays the majority of MAS work uses DNA-based marker, especially after the proliferation of the genome-wide studies due to the lower cost of the genome sequencing strategies. Nevertheless, this markers can be also morphological, biochemical, or cytological. Some of these aspects will be discussed below.

## Antiviral immune response in turbot

### General considerations

In spite of the relevance of turbot culture and the associated pathological process, until a few years ago the knowledge about its immune system was still very fragmentary and little was known about host–pathogen interactions. The pathways implicated in the response against pathogens remained incomplete in turbot and the understanding of how those defense mechanisms act is a relevant factor in order to enhance resistance of cultured fish to diseases. Fortunately, during the last years a great increase in the knowledge of the immune-relevant genes in *S. maximus* has been achieved due to the next-generation sequencing (NGS) strategies (Pereiro et al., 2012a; Ribas et al., 2013; Ma et al., 2016). Indeed, in a 454-pyrosequencing conducted using tissues from turbot inoculated with viral stimuli, including VHSV, the main reference immune pathways of vertebrates (complement pathway, Toll-like receptor signaling pathway, B- and T-cell receptor signaling pathways, and apoptosis) were found to be almost completed with sequences for the genes implicated therein (Pereiro et al., 2012a). But the ultimate step was recently achieved with the publication of the whole genome of turbot (Figueras et al., 2016). With all this information, intensive transcriptome analysis and/or identification of candidate genes implicated in the resistance against a certain disease can be conducted.

### Transcriptome analysis of turbot against VHSV

Several immune genes have been found to be modulated in turbot after VHSV challenge when they were individually analyzed. Nevertheless, some microarray analysis works were conducted, revealing larger and very valuable transcriptome information (Díaz-Rosales et al., 2012; Pereiro et al., 2014b).

Humoral growth inhibitors can act inhibiting the proliferation of the microorganisms by restricting the availability of some elements essential for their proliferation or interfering with their metabolism. Proteins implicated in iron homeostasis, such as transferrin, hepcidin, or hemopexin, acts as a growth inhibitor of bacteria by chelating available iron essential for the bacterial maintenance (Hugman, 2006; Tolosano et al., 2010; Gkouvatsos et al., 2012) or for viral replication (Drakesmith and Prentice, 2008). Two hepcidin genes were described in some fish species, including *S. maximus* (Pereiro et al., 2012b). Both isoforms were modulated after VHSV infection, with down-regulations at short times post-challenge and over-expressions after 24 and 72 h. One of them (*hepcidin-2*) seems not to be implicated in the iron homeostasis, although hepcidins are also related with other immune functions, being a powerful antimicrobial peptide and an immunomodulator in vertebrates (Krause et al., 2000; De Domenico et al., 2010; Rajanbabu et al., 2010). It is interesting to highlight that in a previous work conducted by Díaz-Rosales et al. (2012) using microarrays to compare two low mortality families and two high mortality families of turbot against VHSV, they found that *hepcidin-2* (annotated as *antimicrobial peptide precursor*) was significantly higher expressed in naïve condition in those families showing low mortality rate and therefore, this gene could be directly related with the resistance to the virus. On the other hand, both hepcidin genes were significantly lower expressed at 24 h post-challenge in resistant families compared to susceptible families (Díaz-Rosales et al., 2012). This was also observed in the case of one hemopexin member. Some studies in mice showed that hemopexin, in addition to its role in iron homeostasis, it is also an inflammatory regulator (Liang et al., 2009; Bakker et al., 2010; Spiller et al., 2011). Teleost possess two *hemopexin* (or *warm-acclimation temperature protein*—*wap65*) genes, which were also identified in turbot (Díaz-Rosales et al., 2014). Interestingly, both genes were down-regulated in response to a VHSV challenge; this was probably due to the anti-inflammatory effect of these proteins, which was observed for the first time in teleost by Díaz-Rosales et al. (2014). Indeed, turbot families showing higher resistance to the virus presented a higher inhibition of *wap65-2* expression after the viral challenge (Díaz-Rosales et al., 2012), probably because its role as anti-inflammatory protein diminish the antiviral response.

In this microarray comparing families of turbot with different susceptibilities to VHSV other gene resulted very interesting, the *nk-lysin*. This gene was found to be more expressed in low mortality families in basal conditions (Díaz-Rosales et al., 2012). Nk-lysin (or granulysin in humans) is an antimicrobial peptide produced by NK-cells and cytotoxic T-lymphocytes and stored in cytolytic granules together with perforin and granzymes (Andersson et al., 1995). The perforin–granzyme pathway induce apoptosis of target cells when both molecules are released after the recognition of antigens via major histocompatibility complex (MHC) class I presentation (Trapani and Smyth, 2002). Nevertheless, the function of Nk-lysin in this defensive mechanism is not clear. This peptide was isolated from several vertebrate species and showed a broad antibacterial spectrum (Andersson et al., 1995; Stenger et al., 1998; Lee et al., 2014; Zhang et al., 2014), but its implication in the antiviral response remains to be elucidated. Only a few works were published relating teleost Nk-lysin and viral diseases, but mainly based in its gene induction after infection (Zhang et al., 2013; Pereiro et al., 2015). Zhang et al. (2013) characterized one *nk-lysin* gene in other flatfish, tongue sole (*Cynoglossus semilaevis*), and their results revealed that the intramuscular (i.m.) injection of an expression plasmid encoding this peptide was able to reduce the proliferation of the megalocytivirus in kidney and spleen, as well as to modulate several immune genes, but protection results were not provided. After that, the same authors used a synthetic peptide derived from the tongue sole Nk-lysin and observed that the intraperitoneal (i.p.) injection of this molecule also had an effect in megalocytivirus replication and in the modulation of some immune-related genes (Zhang et al., 2014). As was exposed at the 13th Congress of the International Society of Developmental and Comparative Immunology (ISDCI), some experiment conducted in our laboratory confirmed the link between turbot Nk-lysin and the higher resistance to VHSV (Smith et al., 2016). This relationship is still under investigation, but Nk-lysin could be a promising resistance marker for selective breeding.

Other growth inhibitors are some of the numerous genes induced by type I interferon (IFN), this is interferon-stimulated genes (ISGs), which can reduce the viral proliferation in the host using different blocking mechanisms or strategies (Sadler and Williams, 2008; Varela et al., 2014a). For that reason, the IFN system is considered the main antiviral immune process in vertebrates. The first characterizations of type I IFNs in teleost were conducted in 2003 for zebrafish (Altmann et al., 2003), Atlantic salmon (Robertsen et al., 2003) and pufferfish (Lutfalla et al., 2003) and since that, type I IFNs have been identified in several fish species (Zou and Secombes, 2011). Teleost possess multiple copies of IFN genes in the genome which can present different immune properties, suggesting complementary or specialized roles (Zou et al., 2007; Aggad et al., 2009; López-Muñoz et al., 2009). Two type I IFNs (*ifn1* and *ifn2*) were recently characterized in *S. maximus* and, as was expected, a different action mechanism was observed (Pereiro et al., 2014a). Whereas, the injection of an expression plasmid encoding *ifn1* was able to significantly reduce the mortality rate of turbot against VHSV by diminishing the viral replication, *ifn2* did not show any protective ability against the virus (Pereiro et al., 2014a). Gene expression analysis revealed that only *ifn1* induced the expression of ISGs, whereas the activity of *ifn2* was more restricted to an immunomodulatory function (Pereiro et al., 2014a). Therefore, *ifn1* could serve as a good antiviral treatment of VHSV-infected individuals, helping to eradicate an outbreak in a turbot farm.

The most exhaustive analysis of the immune response in turbot after VHSV challenge was conducted using a microarray enriched in antiviral sequences (Pereiro et al., 2014b). This microarray was used for analyzing both the gene modulation after the administration of a DNA vaccine specific for VHSV and the transcriptome profiles after VHSV challenge in vaccinated and non-vaccinated fish. As was mentioned above, the vaccine encoding the G glycoprotein induced a very similar response to that produced by the virus itself; this is high activation of the main immune pathways (toll-like receptor signaling pathway, IFN system, apoptosis, death induced by cytotoxic cells, MHC-I antigen presentation process, etc.). All these results could provide valuable information for further investigations about the host–pathogen interaction. It is interesting to highlight, among other remarkable modulations, that TLRs with a typical role in bacterial component detection (*tlr5* and *tlr6*) were found to be strongly overexpressed after VHSV infection, and this could suggest a novel role of these receptors in the recognition of viral components (Pereiro et al., 2014b). The coagulation and complement cascades, which are intimately related, were also affected by the virus administration. VHSV is a hemorrhagic virus causing widespread hemorrhages in fish tissues, which induces inflammation and the modulation of pro-coagulant and anti-coagulants factors (Pereiro et al., 2014b). The complement system, composed of more than 35 proteins, is organized into a cascade that starts with the identification of pathogenic surfaces and finally produces the targeted lysis of the pathogen through the membrane attack complex (MAC; Dunkelberger and Song, 2010). Additionally, the activation of this system also induces the generation of proinflammatory mediators and opsonization of the pathogen surface (Dunkelberger and Song, 2010). The central component of the complement system is the component C3, which is proteolytically activated through the classical (through antibody binding), lectins or alternative pathways (in both cases an antibody independent activation). VHSV challenge increased the expression of the majority of the analyzed genes but, interestingly, a significant reduction in the transcription of *complement C1q subcomponent subunit C* was observed, and also a more discrete inhibition in the level of *mannanan-binding lectin serine protease-2* (Pereiro et al., 2014b). This probably indicates a prioritization of the alternative pathway at short times after VHSV infection. Indeed, the transcription profile after infection in vaccinated individual (presenting specific anti-VHSV antibodies) showed an up-regulation of both genes, the first one forming part of the C1 complex of the classical pathway of the complement, and the second one implicated in the lectin pathway.

It is interesting to highlight that, besides the potential implication of these immune-related genes in the defense mechanisms against VHSV, numerous genes with an unknown function or a well-described role in other biological or molecular processes not directly related to the immunity, were also modulated after VHSV challenge (Díaz-Rosales et al., 2012; Pereiro et al., 2014b). This opens the door to further lines of investigation, in which new antiviral genes could be identified.

### Effect of VHSV in immune cells

Macrophages are one of the main immune cells acting against viral infections (Mogensen, 1979). Their ability to phagocyte, to produce substances highly toxic for microorganisms as reactive oxygen species (respiratory burst) and nitric oxide (NO), and the production and release of numerous molecules implicated in the antiviral defense, are the main mechanisms used by this cell type to combat viruses (Murray and Wynn, 2011).

Almost 20 years ago it was determined that VHSV was able to infect and replicate in turbot head kidney macrophages and blood leukocytes primary cultures both from turbot (*S. maximus*) and rainbow trout (*O. mykiss*), although no cytopathic effect was observed and the viral titers were lower than those obtained in the fish cell lines (Tafalla et al., 1998). In these *in vitro* assays, the respiratory burst activity was measured at several days post-infection, but no significant differences were observed comparing with the control groups (Tafalla et al., 1998). On the other hand, an increase of nitric oxide (NO) production was found in turbot kidney macrophages after VHSV challenge, and an exogenous supplementation of NO had an antiviral effect on VHSV, significantly inhibiting its replication (Tafalla et al., 1999). Moreover, other *in vitro* assays conducted in turbot macrophages revealed that VHSV was able to significantly reduce the production of NO induced by tumor necrosis factor α (TNF-α) and macrophage-activating factor (MAF; Tafalla et al., 2001). When *in vivo* VHSV infections where conducted, increases in the macrophage respiratory burst were observed, although the production of NO was not found to be affected (Tafalla et al., 2001). Even the administration of sera from VHSV-infected turbot was able to significantly induce macrophage respiratory burst *in vitro* (Tafalla et al., 2001).

Little is known about the interaction between host cells and VHSV. A recent publication revealed that the spring viremia of carp virus (SVCV), which is also a Rhabdovirus, is not only able to infect zebrafish (*Danio rerio*) macrophages *in vivo*, but these are, at least during the first stages of infection, its target cells (Varela et al., 2014b). During the first hours of infection in zebrafish larvae, the number of macrophages is substantially reduced due to a process of cell death, more specifically through virus-induced pyroptosis of macrophages (Varela et al., 2014b). Probably this process is also occurring in turbot after VHSV challenge. Indeed, microarray analysis revealed a reduction in the expression level of the *macrophage receptor with collagenous domain* (*marco*) and the *macrophage mannose receptor 1* (*mrc1*), two phagocyte receptor molecules that recognize bacterial components, after VHSV challenge in head kidney samples (Pereiro et al., 2014b). Moreover, *macrophage colony-stimulating factor 1* (*csf1*) and *precursor of second macrophage colony stimulating factor* (*csf1-2*), implicated in the macrophage proliferation and differentiation, were also down-regulated (Pereiro et al., 2014b); this was also observed in the case of *interleukin-18* (*il18*), a pro-inflammatory cytokine mainly produced in macrophages and typically highly expressed after viral stimuli (Boraschi and Dinarello, 2006). On the contrary, the cell marker associated to dendritic cells, *cd83*, was strongly overexpressed in response to VHSV (Pereiro et al., 2014b). This dendritic cell marker can also be expressed on the surface of macrophages (Zhou and Tedder, 1996) and neutrophils (Iking-Konert et al., 2002) under certain inflammatory conditions, and as consequence they evolve into functionally mature CD83+ dendritic cells. The reduction in the expression of the genes associated to macrophages after VHSV challenge could let us think in three hypotheses: the destruction of macrophages by VHSV as occurs in zebrafish with SVCV, the reduction of the number of these cells as a defense mechanisms for avoiding the fast replication of this virus in macrophages, or the transformation of the macrophages into functional dendritic cells. More investigations will be needed in order to clarify this question. Regarding to other immune cells, a slight up-regulation of cytotoxic T-cell markers was observed in these first stages of infection, whereas several T-cell receptor (TCR) chain regions and B-cell receptor (BCR) associated proteins were down-regulated (Pereiro et al., 2014b).

### Antibodies production

The presence of specific antibodies against the G glycoprotein in turbot sera was observed 1 month after the administration of a highly effective DNA vaccine encoding this viral protein (pMCV1.4-G_860_), whereas no specific antibodies were detected in the sera from control turbot (PBS or pMCV1.4-injected fish; Pereiro et al., 2012c). This was determined through an enzyme-linked immunosorbent assay (ELISA) using a synthetic peptide from the glycoprotein of the VHSV 07.71 as antigen (Chico et al., 2010), and a monoclonal antibody to turbot IgM (Estevez et al., 1994). Moreover, these antibodies showed neutralizing activity against the virus (Pereiro et al., 2012c). The production of specific and neutralizing antibodies against VHSV was also largely observed in rainbow trout (*O. mykiss*) after VHSV challenge or immunization using viral proteins or DNA vaccines (Lorenzen et al., 1993; Fregeneda-Grandes and Olesen, 2007; Martinez-Lopez et al., 2013).

## Marker-assisted selection (MAS)

In aquaculture industry, there are mainly two desirable traits to improve production: growth and resistance to diseases. Nowadays, the MAS of productive traits is mainly based in DNA markers, which can be used to detect allelic variation in the genes underlying a certain trait (Collard et al., 2005). Therefore, the selection is not based on the trait itself, but on the marker linked to it. In this way, the resistance to fish diseases could be improved by using DNA markers to assist in turbot breeding, and this consists in the selection of allelic variations that are linked with the resistance to diseases. The traits are usually controlled by several genes and are known as quantitative traits (Collard et al., 2005). The quantitative trait loci (QTLs) are those regions of the genome containing genes related with a quantitative trait, and the construction of physical linkage maps makes possible to identify these chromosomal regions (Mohan et al., 1997). The marker used for selection is associated in a high frequency with the QTL of interest due to the proximity on the chromosome and therefore they should co-segregate (genetic linkage; Mohan et al., 1997).

Numerous QTLs associated with the resistance and to VHSV have been identified in *S. maximus* (Rodríguez-Ramilo et al., 2014). Previously to this, QTL analysis were also studied to identify those regions associated with the resistance to the bacteria *Aeromonas salmonicida* (Rodríguez-Ramilo et al., 2011) and the parasite *Philasterides dicentrarchi* (Rodríguez-Ramilo et al., 2013). The existence of an accurate linkage map in turbot (Bouza et al., 2007) was crucial in the detection of these QTLs. Some QTLs were found to be related with the resistance to more than one pathogen (Rodríguez-Ramilo et al., 2014), which is very interesting for designing selective programs. Until the sequencing of the turbot genome (Figueras et al., 2016), the identification of candidate genes associated to the genetic markers was mainly based on comparative mapping between the turbot genetic map and the genome of model teleost species by analyzing syntenic areas (Rodríguez-Ramilo et al., 2014). At the present time, the whole genome sequencing of turbot has led to identify, in a reliable and robust manner, numerous candidate genes associated with the resistance to VHSV (Figueras et al., 2016). The genetic markers were located on the genome and gene mining analysis around the QTLs was conducted using a ±1 Mb window and, among the more than 200 candidate genes identified for VHSV, some of the most remarkable finding were numerous genes implicated in T-cell activity, blood coagulation cascade (probably due to the hemorrhagic activity of this virus), and genes related to iron homeostasis and scavenging (like some transferrin-related genes and *hepcidin*; Figueras et al., 2016). As was mentioned above, *hepcidin* genes were previously associated with resistance to VHSV in turbot families using microarray analysis (Díaz-Rosales et al., 2012). With all this new information available for the researches, the knowledge about the genes implicated in the defense against this viral disease will be probably expanded in the next years.

## Conclusions

In summary, this review tried to comprise all the information available about the immune interaction between *S. maximus* and VHSV, which has not been extensively studied. Hitherto this viral disease is still a serious threat in the turbot farms due to the absence of vaccines or effective commercial treatments. A thorough knowledge of the host–pathogen interaction as well as the identification of those molecules directly implicated in the defense against VHSV are the key points that need to be addressed in order to reduce the prevalence of this disease. Nowadays, both the identification of potential treatments based in self-molecules from turbot and the genetic improvement of the breeders are the main strategies under investigation.

## Author contributions

PP, AF, and BN have conceived, drafted, and approved the final version of this review.

## Funding

This research in our group is funded by projects AGL2014-51773-C3-2-R from Ministerio de Economía y Competitividad, the European project PARAFISHCONTROL (634429), and the “Proyecto Intramural Especial, PIE” (201230E057) from Agencia Estatal Consejo Superior de Investigaciones Científicas (CSIC).

### Conflict of interest statement

The authors declare that the research was conducted in the absence of any commercial or financial relationships that could be construed as a potential conflict of interest.
